# Relationship between admission criteria and academic performance in basic science courses in health science colleges in KAU

**DOI:** 10.1186/s12909-021-02502-4

**Published:** 2021-02-08

**Authors:** Aliaa Amr Alamoudi, Hind Ibrahim Fallatah, Basmah Medhat Eldakhakhny, Fatemah Omar Kamel, Lana Adey AlShawwa, Ayman Zaky Elsamanoudy

**Affiliations:** 1 Department of Clinical Biochemistry, Faculty of Medicine, King Abdulaziz University, Jeddah, Saudi Arabia; 2 Department of Medical Education, Faculty of Medicine, King Abdulaziz University, Jeddah, Saudi Arabia; 3 Gastroenterology Unit, Department of Medicine, King Abdul Aziz University Hospital, Jeddah, Saudi Arabia; 4 Department of Pharmacology, Faculty of Medicine, King Abdulaziz University, Jeddah, Saudi Arabia

**Keywords:** Admission exams, Aptitude, Achievement, Preparatory year, Clinical-Biochemistry, Clinical-Pharamcology, King Abdulaziz University

## Abstract

**Background:**

At King Abdulaziz University, medical and health science schools depend on admission exams (aptitude and achievement) and preparatory year scores in their students’ selection. However, with the growing number of applicants and the drastic changes in teaching and assessment in these colleges, continuous assessment and development of admission criteria are needed. In this study, we aimed to evaluate the correlation of admission exam scores, in addition to the preparatory year Grade Point Average (GPA), with academic performance in the basic science subjects such as Clinical Biochemistry and Clinical Pharmacology in health science colleges.

**Methods:**

The study was conducted on four cohort studies, two faculty of nursing cohorts; nursing students (2017-2018, *n*=146) nursing students (2018-2019, *n*=81), and two faculty of applied medical sciences cohorts, clinical nutrition students (2017-2018, *n*=33), and clinical nutrition students (2018-2019, *n*=28). The students’ scores of General Aptitude Test (GAT), Scholastic Achievement Admission Test (SAAT), and preparatory year GPA were all recorded at the beginning of each semester before the beginning of courses. Clinical Biochemistry and Clinical Pharmacology exam results were recorded at the end of the semester. Correlation was done for each cohort and all cohorts pooled.

**Results:**

Results showed only a weak correlation detected between SAAT and the overall achievement in Clinical Biochemistry (*r*= 0.192, *P*= 0.042) in nursing students (2017-2018), but no correlation was seen with SAAT or preparatory year scores. There was also no significant correlation between admission exams scores and the students’ academic achievement in Clinical Biochemistry or Clinical Pharmacology. On the other hand Clinical Pharmacology exam results showed a significant positive correlation with Clinical Biochemistry results (*r*=0.688, *P*=0.000).

**Conclusion:**

Our results could indicate the need to revisit the admission criteria for these colleges. Furthermore, specific preparatory year tracks for health science colleges can ensure that students improve the specific skills and knowledge required for their future college years3

## Background

Pre-admission criteria have been introduced in medical schools and health sciences colleges worldwide to help enroll students with the highest standards that are more likely to succeed in the field [1–3]. Such criteria are often in the form of academic achievement such as cumulative high school scores, and achievement tests set to assess cumulative knowledge acquired throughout the final years in school. Other cognitive tests that can measure analytical, deductive, and problem-solving skills, in addition to non-cognitive performances, such as multiple mini-interview (MMI), are also usually involved in various medical schools [1–3]. The weight given to each criterion is different between various medical and health sciences schools and remains to be under continuous assessment and development to ensure a meticulous filtration with a growing number of applicants.

In Saudi Arabia, medical and health science schools are no different and depend on pre-admission variables in their students’ selection. These include the cumulative high school average, the Scholastic Achievement Admission Test (SAAT) (known as Tahsili), and the General Aptitude Test (GAT) (known as Qudrat). In addition to the mentioned criteria, at King Abdulaziz University (KAU), students need to finish a preparatory year before being able to enroll in their selected colleges. After finishing all preparatory year courses, students can choose to apply to their desired colleges if their GPA (which is calculated based on a specific formula) meets the requirements of the selected college. In recent years, there has been much controversy on whether the preparatory year achieves some of its goals such as adjusting admission to guide students to the appropriate college that matches students’ abilities and skills, and prepares students for their subsequent colleges. Furthermore, this has been the trigger for the call of establishing more defined tracks in the preparatory year, such as a health colleges’ track. In addition, there has been great interest in assigning weight on non-academic assessments such as the current MMI.

A few studies, the majority being in Riyadh, have evaluated the predictive validity of the Saudi national pre-admission criteria (cumulative high school average, SAAT, GAT) on health science or medical students’ performance [4–6]. This was done through assessing the cumulative GPA whether during their early performance in the first semester or at the end of their pre clinical and/or clinical years. To date, however, there have been no published studies investigating the role of KAU preparatory year for predicting academic success in health science colleges or specifically for predicting success in basic science courses given during the early years of college.

The aim of this study was to assess the correlation of admission criteria (the SAAT and GAT exams) to health science colleges, in addition to the preparatory year GPA, with academic performance in the basic science subject of Clinical Biochemistry. To further validate our results, the study also assessed the correlation with another basic science subject Clinical Pharmacology in one of the cohort groups.

## Methods

### Participants

The study was performed in KAU and included second-year nursing students, Faculty of Nursing, and second-year clinical nutrition (C. Nutrition) students, Faculty of Applied Medical Sciences. The study included data of four cohorts; two faculty of nursing cohorts; nursing students (2017–2018) referred to as Nursing 1 (*n* = 146 ), nursing students (2018–2019) referred to as Nursing 2 (*n* = 81), and two faculty of applied medical sciences cohorts, C.Nutrition students (2017–2018) referred to as C.Nutrition 1(*n* = 33), and C.Nutrition students (2018–2019) referred to as C.Nutrition 2 (*n* = 28). All students were female as there are no male students in either faculty, in addition they were all Saudi Arabian, as only Saudi students are allowed to be enrolled in KAU health science colleges. Students’ age ranged between 19 and 21 years old (19.5 ± 0.56) with no significant differences between all 4 groups. In addition, all students had their high school certificate as their highest level of education. All 4 groups have joined the same science track of preparatory year and thus have undergone the same courses prior to their enrollment in their new college.

### Admission criteria and exams’ records

The students’ scores of GAT, SAAT, and preparatory year GPA were all recorded at the beginning of each semester before the onset of courses. Clinical Biochemistry and Clinical Pharmacology exam results were recorded at the end of the semester. A summary of the exams is mentioned in the sections below.

### General Aptitude Test

The GAT was established to use mathematical and verbal skills to measure: (1) Reading comprehension; (2) Logical relations; (3) Problem solving skills; (4) Inductive skills; (5) Deductive skills. The test is scored out of 120 and is divided with verbal and quantitative questions (Table 1).

**Table 1:** General Aptitude test questions distribution

Verbal	Quantitative
**68 questions**	**52 questions**
Sections include: -Reading comprehension -Sentence completion -Verbal analogy -Synonyms	Sections include: - Arithmetic (40 %) - Geometry (24 %) - Algebra (23 %) - Statistics and analytical questions (13 %)

Overall, the test aims to measure the student’s capacity for learning in general rather than measuring a specific skill required for specific topics or fields.

Students are allowed to take the GAT more than once. However, it is expected that students’ scores do not change drastically with further trials. Furthermore, students can use any of the GAT scores obtained to apply for university.

### Scholastic Achievement Admission Test (SAAT)

The SAAT test measures the overall achievement of students during their education journey. The test covers key concepts in Biology, Chemistry, Physics, Maths and English in the form of multiple choice questions (MCQ), which are divided equally between the five subjects. The test is scored out of 100 and comprises questions that address: Comprehension, application, inference, and other aspects.

### Preparatory year

In KAU, preparatory year consists of two tracks; a scientific track, and a literature track. The scientific track serves those who wish to join Medicine, Dentistry, Nursing or Applied Medical Sciences, and consists of Math, Chemistry, Biology, Physics, Communication skills, Computing and information technology and Academic English language. After finishing all preparatory year courses, students can choose applying to their desired colleges if their GPA meets the requirements of the selected college.

### Clinical Biochemistry course and exam

During their second year, both groups receive the same course of Clinical Biochemistry (BCHM 207) during their first semester which covers basic cell metabolism, bioenergetics, and molecular biology. The course is given in the form of traditional lectures, tutorials and practical sessions. The 100 final score is divided between two quizzes, a practical exam, and a final exam. All exam questions were in the form of MCQs that are divided into recall questions (70 %) and reasoning questions (30 %).

### Clinical pharmacology

Third-year nursing students were divided into two groups; both groups received the same course of Pharmacology (PHAN311) during their first semester. The course consists of general pharmacology, autonomic nervous system, cardiovascular, renal, endocrine systems and autacoid. Students learned about how drugs act on the covered systems, the use of the drug in treating various diseases, its adverse effects and possible interactions with other drugs taken at the same time. The course was given in the form of scheduled lectures, self-directed learning, and tutorials, which ensure smooth flow of the scientific material in a controlled manner through several pathways to achieve course objectives. The 100 final scores was divided between mid-block exam, assignment, and a final exam. All exam questions were in the form of MCQs, divided into recall questions (70 %) and reasoning questions (30 %).

### Statistical analysis

All analysis was carried out using GraphPad Prism Version 8.3.1 (332). Descriptive analyses were described as mean ± standard deviation (SD), minimum and maximum scores. Analysis of variance (ANOVA) with post hoc test was used for testing the significance between groups. Pearson correlation coefficient was used to assess the correlation between the different exam results. *p* < 0.05 was taken as a cut off value for significance.

## Results

### Preparatory year GPA, GAT, SAAT, and clinical Biochemistry results in all cohorts

**Fig. 1:**
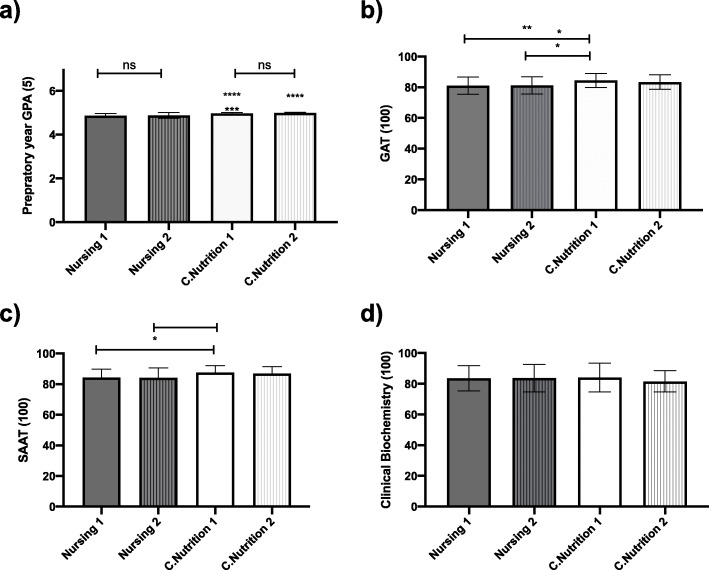
Scores for a) Preparatory year b)GAT c) SAAT d) Clinical Biochemistry for Nursing (dark bars) and C. Nutrition 1 & 2 (light bars). Error bars represents mean and SD. **** represents *P* < 0.0001 between preparatory year scores in Nursing 1 and C. Nutrition 1 & 2, and Nursing 2 and C. Nutrition 2 .*** represents *P* < 0.001 between preparatory year scores in Nursing 2 and C. Nutrition 1. ** represents *P* < 0.01 between GAT scores in Nursing 1 and C.Nutrition 1. * represents *P* < 0.05 between GAT scores in Nursing 2 and C.Nutrition 1. In addition * represents *P* < 0.05 between SAAT scores in Nursin 1 & 2 and C.Nutrition 1

Descriptive statistics of all four cohorts are described in Table 2. There was no significant difference between the preparatory year GPA between the two nursing cohorts, or the two C.Nutrition cohorts (Fig. 1). However, C.Nutrition cohorts showed a significantly higher GPA than the nursing groups (Fig. 1). C. Nutrition 1 result in GAT (84.44 ± 4.52) was significantly higher than Nursing 1 81.01 ± 5.58, *P* < 0.01) and Nursing 2 (81.18 ± 5.588, *P* < 0.05). While C.Nutrition 2 GAT results (83.38 ± 4.79) were higher than both nursing groups, this did not reach significant levels. A similar pattern was seen with SAAT results, in which C. Nutrition 1 results (87.55 ± 4.47) were significantly higher than Nursing 1 (84.35 ± 5.47, *P* < 0.05) and Nursing 2 (84.09 ± 6.51, *P* < 0.05).

On the other hand, there was no significant difference between all groups in their Clinical Biochemistry exam results.

**Table 2:** Descriptive statistics of Preparatory year GPA, GAT, SAAT and Clinical Biochemistry results

Cohort Statistics	Nursing 1 (146)	Nursing 2 (81)	C. Nutrition 1 (33)	C. Nutrition 2 (28)
Preparatory year (GPA)				
Mean	4.86	4.87	4.96	4.98
Minimum	4.15	4.30	4.82	4.90
Maximum	5.00	5.00	5.00	5.00
Std. Deviation	0.09	0.13	0.04	0.02
GAT (score)				
Mean	81.01	81.18	84.44	83.38
Minimum	65.00	68.00	74.00	75.00
Maximum	95.00	95.00	92.00	93.00
Std. Deviation	5.58	5.58	4.52	4.79
SAAT (score)				
Mean	84.35	84.09	87.55	86.88
Minimum	69.00	66.00	79.00	74.00
Maximum	97.00	99.00	97.00	94.00
Std. Deviation	5.47	6.51	4.47	4.52
Clinical Biochemistry (score)				
Mean	83.62	83.11	84.06	81.54
Minimum	60.00	60.00	61.00	68.00
Maximum	99.00	97.00	100.00	97.00
Std. Deviation	8.20	10.26	9.41	6.95
Clinical Pharmacology (score)				
Mean	75.63			
Minimum	47.00			
Maximum	95.00			
Std. Deviation	10.61			

### Correlation between Clinical Biochemistry exam results and preparatory year GPA, GAT, and SAAT results

There was no significant correlation between Clinical Biochemistry exam results and preparatory year GPA, GAT, or SAAT results in all cohorts except for Nursing 1 Table 3. In this cohort, a significant positive correlation was seen between Clinical Biochemistry and SAAT (*r* = 0.192, *P* = 0.042), with r^2^ (coffeicent of determination) = 0.036, indicating that only 3 % of variation in Clinical Biochemistry scores could be explained with variations in SAAT .

Similarly, there was no significant correlation between preparatory year GPA and GAT or SAAT results in all cohorts except for Nursing 1. In this cohort, a significant correlation was seen between the preparatory year GPA and GAT (*r* = 0.211, *P* = 0.024).

However, a significant positive correlation was seen between GAT and SAAT results in all cohorts; Nursing 1 (*r* = 0.406, *P* = 0.000), Nursing 2 (*r* = 0.499, *P* = 0.000 ), C.Nutrition 2 (*r* = 0.468, *P* = 0.016), except for C. Nutrition 1 in which the correlation did not reach significant results (*r* = 0.305, *P* = 0.090).

When combining all 4 cohorts, we did not see any significant correlation between Clinical Biochemistry results and preparatory year GPA (*r* = 0.026, *P* = 0.679), GAT (*r* = 0.028, *P* = 0.659) or SAAT (*r* = 0.068, *P* = 0.280) results.

**Table 3:** Correlation between Clinical Biochemistry exam results and preparatory year GPA, GAT, and SAAT results

	Preparatory year	GAT	SAAT
Nursing 1			
Clinical Biochemistry	*r*=0.017	*r*=-0.047	*r*=0.192
	*P*=0.857	*P*=0.624	*P*=0.042
Preparatory year		*r*=0.211^*^	*r*=0.146
		*P*=0.024	*P*=0.119
GAT			*r*=0.406
			*P*=0.000
Nursing 2			
Clinical Biochemistry	*r*=0.042	*r*=-0.112	*r*=0.138
	*P*=0.708	*P*=0.317	*P*=0.218
Preparatory year		*r*=0.190	*r*=0.147
		*P*=0.072	*P*=0.167
GAT			*r*=0.499
			*P*=0.000
C. Nutrition 1			
Clinical Biochemistry	*r*=0.309	*r*=-0.230	*r*=0.211
	*P*=0.086	*P*=0.213	*P*=0.246
Preparatory year		*r*=0.289	*r*=-0.112
		*P*=0.108	*P*=0.534
GAT			*r*=0.305
			*P*=0.090
C. Nutrition 2			
Clinical Biochemistry	*r*=0.139	*r*=0.131	*r*=0.024
	*P*=0.549	*P*=0.522	*P*=0.906
Preparatory year		*r*=-0.063	*r*=-0.086
		*P*=0.785	*P*=0.712
GAT			*r*=0.468
			*P*=0.016

### Correlation between Clinical Pharmacology exam results and preparatory year GPA, GAT, SAAT, and Clinical Biochemistry exam results

When analysing Clinical Pharmacology exam results in Nursing 2, there was no significant correlation with Preparatory year, GAT or SAAT results in Table 3. Clinical Pharmacology exam results, on the other hand, showed a significant positive correlation with Clinical Biochemistry results in Nursing 2 (*r* = 0.688, *P* = 0.000) Table 4.

**Table 4:** Correlation between Clinical Pharmacology exam results and preparatory year GPA, GAT, SAAT, and Clinical Biochemistry exam results

	Preparatory year	GAT	SAAT	Clinical Pharmacology
Clinical Biochemistry	*r* = 0.017 *P* = 0.857	*r*=-0.047 *P* = 0.624	*r* = 0.192 0.042	*r* = 0.688^**^ *P* = 0.000
Clinical Pharmacology	*r*=-0.061 *P* = 0.584	*r*=-0.024 *P* = 0.830	*r* = 0.039 *P* = 0.727	

## Discussion

In medical and health science schools, the selection of the right students is considered a challenge that requires a lot of prerequisites and efforts. El Says et al., [7] summarized the admission requirement and specified the importance of each requirement regarding the Faculty of Medicine-KAU (FOM-KAU). Students’ scores in high school, standard achievement admission tests (SAAT and GAT), as well as their scores in preparatory year are the measures that can evaluate the cognitive skill of students. GAT is specifically designed to evaluate the student’s general characteristics and gained abilities. GAT also measures the learned skills in critical thinking and the ability of non-verbal problem solving and reflections. On the other hand, SAAT is designed to measure the overall achievement of students during their education journey. Finally, multiple mini-interviews (MMIs) come to assess the non-cognitive aptitude since MMIs can measure some importantly required personal capabilities like professionalism, empathy, and communication skills [8, 9].

Whether these tests and prerequisites for selecting the students to health science schools predict their academic achievement remains to be questionable. Hence, this study aimed firstly to assess the correlation of admission exams (the SAAT and GAT exams) to health science colleges and preparatory years scores in KAU, with academic performance in the basic science subjects such as Clinical Biochemistry and Clinical Pharmacology.

The current study revealed a significant positive correlation between SAAT and GAT results in the programs of the study (Nursing 1& 2 and C.Nutrition 1&2). SAAT and GAT are the eligibility tests that are documented in Saudi Arabia for science and health science schools. These tests are carried out by the National Centre for Assessment in Higher Education (NCAHE)- Ministry of Education. The type and procedure of these fair assessments are MCQ exams, which can be taken as a maximum of three attempts with the best score of those three considered for students’ admission. This procedure could allow a fair evaluation of the students at all levels of education.

Moreover, it is considered an effective eligibility examination for the university graduation programs [10]. It is therefore logical to find a significant positive correlation between SAAT and GAT for students, as documented in the current study. This correlation was also previously reported by Alnahdi [11], and Al-Qahtani & Alanzi [12].

Moreover, in the current study, there was no significant correlation between their admission exams scores and the students’ academic achievement in Clinical Pharmacology, and with only a weak correlation detected between SAAT and the overall achievement in Clinical Biochemistry. Yet, this correlation would not seem to have a strong effect size since only 3 % of the variation in Clinical Biochemistry scores would be explained with variation in the SAAT scores.

The predictive value of standardized admission tests scores for academic achievements remains to be controversial and a matter of debate in several institutes worldwide. Many studies have reported opposing results to the results of the current study as they have reported predictive values of standardized admission tests with academic performance in nursing [13–15]. However, non-conclusive and conflicting findings between these standardized admission tests and the students’ college performance in Saudi Arabia [6] India, and Canada [16] were also reported. For example, in one of the earlier studies in Saudi Arabia conducted at King Saud bin Abdulaziz University for Health Sciences, a strong correlation between the academic performance and the achievement exam, aptitude exam and high school final grade was found; although this was most seen with the achievement exam [5]. However, the study was done on a very small sample of 91 students. In a larger study on 737 students, the SAAT, but not GAT, was a positive predictor of cGPA but only in the pre-clinical years and not clinical years [6].

Such poor association can be explained by the fact that teaching methods and assessment plans in the university programs (Nursing and C.Nutrition programs in the current study) have changed dramatically over the past years and are entirely different from that of high school. The high school teaching strategy depends mainly on the students’ ability to recall data and knowledge while our programs aim at using different teaching and assessment strategies that depend on cognitive achievement rather than recall, such as practical and clinical application, analysis, and critical thinking, scientific problem solving as well as synthesis abilities. Such competencies are usually not fully covered in high schools and might be poorly assessed by the already used eligibility tests, which support the explanations that are previously stated in the literature [11, 12]. Moreover, some non-academic competencies are gained in university programs rather than in high schools. These include leadership, communication skills, community and social services, and interpersonal and teamwork activities [17]. All of these competencies are extracurricular activities that motivate students and enhance their academic achievement and are usually part of the overall assessments in medical and health science schools in KAU.

In this study, a significant positive correlation between the overall results of Clinical Biochemistry and that of Clinical Pharmacology is important as it ensures internal validity, and indicates that the poor correlation between performance and admission criteria might be a feature to all basic science subjects, but this would require further studies to confirm. This result can be explained by the fact that both departments use nearly similar instructional methods and teaching strategies (traditional lecturing, problem-based learning, mini-projects, and students directed learning activities), and follow the same protocols of assessment. Consequently, a positive correlation between the overall results of both (Clinical Biochemistry and that of the Clinical Pharmacology) is a logical finding of the current study.

Given the lack of previous studies in KAU on the matter, investigating the possible correlation between the preparatory year GPA with academic performance in our basic science subjects was our second aim in this study, and indeed no significant correlation was found.

The preparatory year in Saudi universities, including KAU aims to be an educational program that lasts for two semesters. The objectives of the preparatory year are not for purely educational purposes only but also aim to prepare university students at the social, psychological, and cultural levels. It aims to enable them for skillful communication and self-learning ability.

Despite these valuable objectives, students’ satisfaction with the preparatory year is below expected [7]. This lack of satisfaction could be explained by the interplay of students, faculty, and program-related factors [7, 18]. Consequently, failure to meet students’ expectations and achievement of the program objectives is the overall result, which leads to a negative impact on students. This can also result in its failure to be a predictive indicator of students’ academic achievement later on in the subsequent university years, which was seen in our current study. Overall these results could also validate the need for various tracks and programs in KAU preparatory year, which could serve the different colleges’ needs and competencies rather than having a general program for all science colleges.

Most studies have looked at the link between admission criteria and the overall final academic achievement; i.e. final GPA. However, we were keen in this study to evaluate how admission criteria can specifically predict success in basic science courses which are given during the early years. This can, first of all, give us a better reflection of the importance of the admission criteria since in the later years many variables can interfere with interpreting the relationship including the adaptation of students to new teaching stratgies along their academic years. In addition, this can help us predict the value of admission criterias for basic science courses and identifiy gaps which can be addressed.

With a growing number of competency based curriculums in health science colleges worldwide, basic science courses are shifting from being a list of topics and a fixed body of knowledge that needs to be covered towards a more structured experience that should lead to the ability to apply knowledge, to havng strong observations and hypothesis testing, and to acquire problem-solving skills. Further studies would be needed to indicate the sorts of skills required for basic science courses and how it can be incorporated in entry exams and evaluations. The results also encourage the need of a culture shift of introducing various cognitive and non-cognitive tests for medical schools and health sciences colleges’ admission criteria. Such tests need to predict whether the students carry some of the skills that would most likely help them to succeed in the new competency based currciulums set by local and regional accreditation institutes. This is specifically important in a region (the Middle East) where there remains to be challenges in higher education, and where interactive learning in both formal and higher education is still relatively new in application.

There are a few limitations in our study. First of all, the results represent a single institution which might limit its generalisability, specifically that basic science courses can be taught and assessed differently from one institute to another. Second of all, digging into more of the participant characteristic can identify if any of these characteristic can interfere with the findings. An important factor, for example, would be differentiating between public and international schools graduates. In addition, a larger sample size would increase the predictive value of the results.

## Conclusions

In this study, our results show that admission tests and preparatory year scores are not predictive of academic performance in basic science courses in health science colleges. This could indicate the need to revisit the admission criteria for these colleges. In recent years, with the founding of the National Commission for Academic Accreditation and Assessment (NCAAA) Center, which aims at contributing to the enhancement of quality and excellence in higher education institutions through evaluation and accreditation, there has been a shift in teaching and assessment strategies in health science colleges.

More competencies and learning outcomes are being required from graduate students of these colleges. It is therefore important that cognitive and non-cognitive admission instruments can assess these competencies. Furthermore, specific preparatory year tracks for health science colleges can ensure that students improve the specific skills and knowledge required for their future college years.

## Data Availability

Not Applicable.

## Abbreviations

C. Nutrition: Clinical Nutrition
GAT: General Aptitude Test
KAU: King Abdulaziz University
MMI: Multiple mini-interview
NCAAA: National Commission for Academic Accreditation and Assessment
SAAT: Scholastic Achievement Admission Test
SD: Standard Deviation
